# Behavioral interventions related to plastic waste management in low-and middle-income countries: a systematic review using the behavior change wheel and the theoretical domains framework

**DOI:** 10.1088/1748-9326/ae49a3

**Published:** 2026-03-06

**Authors:** Hina Raheel, Annalyse Ferguson, Sharon L Leslie, Vanessa Guardado-Menjivar, Kelsey Chen, Alina Merceron, Jessica Arciniegas, Amy E Lovvorn, Melinda Higgins, Dana Boyd Barr, Eri Saikawa, Margaret A Handley, Lisa M Thompson

**Affiliations:** 1 Nell Hodgson Woodruff School of Nursing, Emory University, Atlanta, GA, United States of America; 2 Choptank Community Health Systems, Denton, MD, United States of America; 3 Woodruff Health Sciences Center Library, Emory University, Atlanta, GA, United States of America; 4 Metropolitan Area Planning Council, Boston, MA, United States of America; 5 Florida Department of Health, Tallahassee, FL, United States of America; 6 Gangarosa Department of Environmental Health, Rollins School of Public Health, Emory University, Atlanta, Georgia, United States of America; 7 Department of Environmental Health Sciences, Emory University, Atlanta, Georgia, United States of America; 8 PRISE Center (Partnerships for Research in Implementation Science for Equity), University of California San Francisco, San Francisco, CA, United States of America; 9 Department of Epidemiology and Biostatistics and Medicine, University of California San Francisco, San Francisco, CA, United States of America; 10 Department of Family Health Care Nursing, School of Nursing, University of California San Francisco, San Francisco, CA, United States of America

**Keywords:** behavior change, plastic pollution, COM-B model, pro-environmental behavior, motivation

## Abstract

Addressing the mounting plastic waste problem requires system-level solutions, along with interventions that promote behavioral change. In low-resource countries, inadequate, if not absent, waste management systems lead to unsafe disposal practices, including open burning. While theory-informed approaches are essential for identifying enablers and barriers to target behavior change, their application is limited in these settings. Given the lack of a theory-driven synthesis of behavioral strategies to address plastic waste, this systematic review aimed to: (1) synthesize behavioral interventions related to plastic waste management in low-resource countries; (2) map these interventions to the behavior change wheel (BCW), using the capability-opportunity-motivation-behavior model, and the theoretical domains framework (TDF); and (3) classify implementation strategies to inform theory-driven intervention design. This review is the first to use the BCW to examine behavioral interventions related to plastic waste management in low-resource countries. Nine bibliographic databases: APA PsycInfo, CINAHL, Embase, Environment Complete, Global Health, GreenFile, Health Source: Nursing Academic, PubMed, and Web of Science Core Collection were searched. We included English-language human studies up to 9 April 2025, that evaluated interventions or policies targeting individual- or community-level behaviors related to plastic waste management in low-, lower-middle, or upper-middle income countries. We excluded studies from high-income countries, and those focused on environmental impacts, industrial or municipal waste streams, ecosystems or animals without human behavioral components, COVID-19-specific waste, or hypothetical modeling without real-life interventions. Forty-three studies met the inclusion criteria. Study quality was assessed using the mixed methods appraisal Tool. Interventions spanned 27 low-resource countries and targeted diverse populations, including schoolchildren, households, market vendors, and community organizations. Education was the most frequent BCW intervention function (76.7%), followed by environmental restructuring, incentivization, persuasion, and training. Mapping revealed that behavioral interventions relied most frequently on the TDF domains of environmental context, knowledge, skills, and social influences. Some domains, such as beliefs about capabilities, reinforcement, and identity, received moderate attention, while appealing to emotion or the use of behavioral regulation, were underutilized. Behavioral interventions for plastic waste management in low-resource countries have predominantly emphasized awareness-raising but insufficiently leveraged other BCW intervention functions and TDF domains. Integration of motivational, emotional, and identity-based strategies alongside structural support can enhance the sustainability of behavior change.

## 1. Introduction

Plastic waste has emerged as one of the defining environmental health challenges of the twenty-first century. The United Nations estimates that 400 million metric tons of plastic are generated annually (Singh and Walker 2024, United Nations Environment Programme 2025), and since 1950, an estimated 9.2 billion metric tons have entered global circulation. If this trend continues, another 12 billion metric tons of plastic waste may be generated by the year 2050 (Singh and Walker 2024). Globally, waste management efforts, such as recycling and waste collection, are failing to process or reduce plastic waste effectively. Despite investments in both technological advancements in recycling infrastructure (such as mechanical and chemical recycling processes) (Alaghemandi 2024, Babaremu *et al* 2024) and behavior-focused initiatives aimed at increasing household and industrial recycling (Popescu *et al* 2019, Walzberg *et al* 2023, da Silva *et al* 2025), global recycling rate of plastic remains stagnant at only 9% (Houssini *et al* 2025).

Accumulated plastic has become a leading cause of widespread pollution. The environmental and public health impacts of the growing accumulation of plastic waste are significant and increasingly evident. Poorly managed plastic waste contaminates land and water, harms marine wildlife through ingestion and entanglement, disrupts ecosystems, and breaks down into microplastics that persist in soils, rivers, and oceans (Dey *et al* 2024, Surela *et al* 2025). As plastic waste weathers, leaches, or is burned, it releases nano- and microplastics and chemical additives into the environment, creating clear pathways for human exposure, primarily through ingestion and inhalation (Cook and Halden 2020). Beyond waste itself, everyday use of plastic products—such as food storage containers, plastic cutlery and dishware, bottled water, and plastic-packaged household chemicals—also contributes to ongoing human exposure.

A growing body of evidence links these exposures to a range of adverse health outcomes, including neurologic, cardiac, respiratory, endocrine, and reproductive effects (Wright and Kelly 2017, Landrigan *et al* 2023, Seewoo *et al* 2023). Together, these environmental and health consequences highlight why reducing plastic waste generation and improving waste management practices are critical for protecting both ecosystems and human health (Cook and Halden 2020).

Although plastic waste is a global challenge, low-, lower-middle, and upper-middle income countries are disproportionately vulnerable (Abahussain *et al* 2025, Olaitan 2025) due to inadequate waste management systems and limited infrastructure (Abahussain *et al* 2025). In the absence of proper containment options, many communities resort to open dumping, uncontrolled landfilling, or burning plastic in open fires (Cruz *et al* 2022, Thompson *et al* 2025). Waste incineration leads to acute and chronic air pollution (both indoors and outdoors) (Aydin *et al* 2024, Kearns *et al* 2024). Among all environmental risks, air pollution poses the greatest health risk, contributing to approximately 7.9 million premature deaths in 2023 (State of Global Air 2023). However, air pollution estimates thus far have not included the household-level contributions from burning plastics. Because plastics burn quickly and release intense heat, plastic waste is commonly used to kindle cooking fires within households (Burning plastic can affect air quality, public health 2022, Bharadwaj *et al* 2026). The human health burden from exposure to household air pollution from plastic used as cooking fuel is unknown (Bharadwaj *et al* 2026).

While infrastructural investment in waste management is essential, individual and community practices like waste sorting, participation in recycling, and elimination of open burning are equally critical for mitigating plastic-related harms. A complex interplay of personal, social, and structural factors shapes behaviors related to plastic waste management. A growing body of evidence suggests that behavior change interventions are significantly more effective when they are developed and implemented using relevant theoretical frameworks designed to address these complexities (Michie *et al* 2008, Michie and Prestwich 2010, Webb *et al* 2010, Bonner *et al* 2021). However, despite this recognition, the integration of theory into the design and delivery of such interventions remains relatively scarce (Davies *et al* 2010, Atkins *et al* 2017).

The behavior change wheel (BCW) offers a structured, theory-driven approach to guide intervention development by identifying influences on behavior and addressing them through targeted interventions across diverse settings. It contains the capability-opportunity-motivation-behavior (COM-B) (Michie *et al* 2011) model and the theoretical domains framework (TDF) (Cane *et al* 2012), both of which provide a coherent conceptual foundation for understanding complex behaviors and designing interventions (Michie *et al* 2014). Within the COM-B framework, behavior is shaped by three interacting components: *capability* refers to an individual’s psychological and physical capacity to perform a behavior, including having the necessary knowledge and skills; *opportunity* encompasses the social and environmental conditions external to the individual that enable or constrain behavior; and *motivation* includes both reflective processes, such as conscious decision-making, and automatic processes, such as emotions and habits, that drive behavioral action. This framing provides a systematic approach to identify the key drivers of behavior and to consider how interventions may influence these drivers to promote sustained change.

The TDF expands the COM-B behavioral influences into 14 domains, which encompass cognitive, social, and emotional influences on behavior (Cane *et al* 2012). ‘*Capability’* includes knowledge, skills, memory, attention and decision processes, and behavioral regulation; *opportunity’* includes environmental context and resources, and social influences; and ‘*motivation’* includes social and professional role and identity, beliefs about capabilities, optimism, beliefs about consequences, intentions, goals, reinforcement, and emotion. By articulating the mechanisms through which individuals interpret, respond to, and act within their environments, the TDF offers valuable insight into why certain behaviors persist and others are resistant to change.

Finally, the BCW incorporates nine broad categories of ‘intervention functions’ that can be implemented strategically to the target behaviors: education, persuasion, incentivization, coercion, training, restriction, environmental restructuring, modeling, and enablement (Michie *et al* 2014). These intervention functions offer a structured typology for guiding the implementation of interventions that seek to influence behavior.

The COM-B model and BCW method have been widely utilized in designing public health interventions, such as infection control (Greene and Wilson 2022), hand hygiene (Caruso *et al* 2025), household air pollution and clean energy transitions (Thompson *et al* 2018, Williams *et al* 2020), but their application to plastic waste management remains limited (Allison *et al* 2022). While prior studies have explored strategies to improve waste management practices, including those targeting plastic waste, only one study systematically mapped these behavioral determinants using a theoretical framework and none have looked specifically at the plastic waste issue in low- and middle-income countries. Although Allison *et al* conducted a meta-analysis using COM-B and the BCW to examine behaviors related to plastic waste, their review included primarily high-income countries (2022). They examined effect sizes across a broad range of contexts and systematically mapped intervention components using COM-B and BCW. However, they did not use the TDF, which may limit insight into more granular psychological determinants of behavior. Moreover, there has been new research published after 2022 that was not captured in this review.

Given that plastic waste is fundamentally an equity issue, disproportionately affecting low-, lower-middle-, and upper-middle-income countries (UMICs) (Abahussain *et al* 2025, Olaitan 2025), there remains a critical need for a focused and theory-driven synthesis in these contexts. The World Bank classifies countries into income groups based on gross national income (GNI) per capita, calculated using the Atlas method. As of 2024, low-income countries (LICs) are those with a GNI per capita of $1135 or less; lower-middle-income countries (LMICs) are those $1136 and $4465; and upper-middle-income countries (UMICs) are those between $4466 and $13 845. For this review, we collectively refer to these three groups as ‘low-resource countries’, defined as those with a GNI per capita below $13 845 (‘How does the World Bank classify countries?—World Bank Data Help Desk’ n.d.). This systematic review is the first to use the BCW and the TDF to examine behavioral interventions related to plastic waste management in low-resource countries.

This review aims to:

1. Identify and synthesize behavioral interventions designed to influence plastic waste management in low-resource countries.
2. Systematically map these interventions to the COM-B components and the TDF domains.
3. Classify the BCW intervention functions that were used to address plastic waste reduction, segregation, and responsible disposal practices, offering evidence-based guidance for future community-level interventions targeted at low-resource countries.

The motivation for this review is informed by ongoing research in rural Guatemala, where an implementation science–guided randomized controlled trial is being conducted to deliver a village-level intervention targeting plastic waste management practices, including the burning of plastic in household fires (Thompson *et al* 2025). This research has highlighted the importance of systematically understanding how behavioral interventions are designed and delivered in comparable low-resource settings and has informed our interest in synthesizing evidence from other contexts.

## 2. Methods

### 2.1. Protocol and registration

This review was filed with the International Prospective Register of Systematic Reviews (PROSPERO 2023 CRD42023440661) in accordance with the Preferred Reporting Items for Systematic Reviews and Meta-Analyses (PRISMA) guideline (Page *et al* 2021).

### 2.2. Eligibility criteria

***Inclusion criteria:***

1. Human studies examining implementation of strategies or interventions focusing on behaviors (including knowledge change as a precursor to behavior change) surrounding plastic waste management, or implementation of a management program or a specific policy (e.g. single use plastic bag ban) in LICs, LMICs, and UMICs, at an individual or local community level perspective. Safe management here is defined as coordinated collection, reuse, repurposing, or recycling of plastics.
2. Studies written in English.
3. Studies that take the form of randomized controlled trials, observational studies, or qualitative studies.
4. Studies published through 9 April 2025.

***Exclusion criteria:***

1. Studies in high-income countries (World Bank definition).
2. Studies assessing environmental impact without evaluation of human behaviors that modify exposure risks (e.g. quantifying plastic trash/bags on shores without any intervention to reduce the problem).
3. Studies assessing municipal solid waste composition analysis, or waste cycles/streams/industrial waste.
4. Studies exclusively modeling macro-level system factors of plastic waste control (e.g. those lacking evaluation of individual or local community-level behaviors and barriers as they relate to the larger system).
5. Studies reviewing animal life and ecosystem impact without any application or connection to human behaviors.
6. Studies confined to COVID-19 pandemic waste (such as medical waste disposal, incineration practices, etc), a period of time when increased plastic use and subsequent medical waste and incineration was widely practiced.
7. Empirical studies that do not test an intervention in a population, instead conducting cross-sectional surveys and modeling outcomes using structural equation modeling, willingness to pay, and discrete choice experiments, or other statistical analyzes.
8. Studies assessing behavioral change based on a hypothetical scenario rather than real-life interventions.

### 2.3. Search strategy

A comprehensive literature search strategy was developed and conducted by an experienced medical librarian (SL) with input from the research team to identify relevant articles. Searches were initially undertaken on 20 June 2023, and re-run on 10 April 2025. Pre-identified sentinel articles were hand searched for keywords relating to the study objectives. The searches combined controlled vocabulary supplemented with keywords related to the concepts of plastics, management of waste, behavior change, and low-middle income countries. Individual country names designated as ‘Low-Income Economies’ and ‘Lower-Middle Income Economies’ were obtained from the World Bank Country and Lending Groups website (‘World Bank Country and Lending Groups—World Bank Data Help Desk,’ n.d.). Nine bibliographic databases: APA PsycInfo, CINAHL, Embase, Environment Complete, Global Health, GreenFile, Health Source: Nursing Academic, PubMed, and Web of Science Core Collection were searched. Full search strategies for each database may be found in supplementary material A.

### 2.4. Data management and selection process

Database search results were imported into Covidence (Covidence systematic review software), and duplicates were removed. The title and abstract were screened by five independent investigators (HR, KC, AM, VG, LT) for eligibility, followed by full-text screening of each paper by two independent reviewers (HR, KC, AM, VG, LT). Conflicts between the reviewers were resolved by discussion.

### 2.5. Data extraction

A standard data extraction template was created to unify the data extraction process (supplementary material B). Data from each chosen manuscript was extracted by pairs of individuals selected from a team of five independent reviewers (HR, KC, AM, VG, LT) using this template. Extracted data items included publication details (author and year), settings (country of study), sample population (sample size, age, race, specific region), study purpose and design, type of intervention implemented, outcomes assessed or results of the study, strengths and limitations of the study, and general comments by the reviewers.

### 2.6. Quality assessment and risk of bias

The methodological quality and risk of bias were assessed using the mixed methods appraisal tool (MMAT) (Hong *et al* 2018). The MMAT is designed to critically appraise studies across various research designs, including qualitative, quantitative, and mixed methods approaches (Hong *et al* 2018). For each study design, the MMAT assesses key domains of methodological rigor and potential bias, including the appropriateness of the study design for the research question, adequacy of sampling and participant selection, measurement validity and reliability, completeness of outcome data, and appropriateness of data analysis. For quantitative studies, additional domains include risk of confounding, fidelity of intervention implementation, and consistency of outcome measurement across participants. For qualitative studies, the MMAT examines the coherence between data sources, data collection methods, analysis, and interpretation. Mixed methods studies are further appraised for the quality of integration between qualitative and quantitative components and the adequacy of each component relative to the study objectives. In MMAT, each domain is classified as low, high or unclear, but does not provide an overall risk of bias estimate across domains. The risk of bias for each study was assessed by a pair of five independent reviewers (HR, KC, AM, VG, LT). If required, a third researcher was consulted to resolve disagreements.

### 2.7. Synthesis method

Given the heterogeneity in study designs, exposures, intervention types, outcome measures, and results, the findings were synthesized and narratively guided by the COM-B and the TDF to extract, categorize, and compare intervention strategies and targeted behavioral domains. The BCW was used to determine the presence of nine key intervention functions used to influence behavior in each study: (1) education, (2) persuasion, (3) incentivization, (4) coercion, (5) training, (6) restriction, (7) environmental restructuring, (8) modeling, and (9) enablement. This allowed for mapping each intervention’s core behavior change strategies. The COM-B model was used to identify capabilities (physical and psychological), opportunities (social and environmental) and motivations (reflective and automatic) that would influence behavior change. The 14-domain TDF was used to identify the specific behavioral domains targeted by the intervention. This included assessing whether interventions addressed: (1) skills, (2) knowledge, (3) memory, attention and decision processes, (4) behavioral regulation, (5) environmental context and resources, (6) social influences, (7) social/professional role and identity, (8) beliefs about capabilities, (9) optimism, (10) beliefs about consequences, (11) intentions, (12) goals, (13) reinforcement, and (14) emotion.

See table 1 for the mapping of the COM-B components and the TDF domains onto the BCW intervention functions. Each study was coded for COM-B components, TDF domains and BCW intervention functions by two independent reviewers (HR, AL and JA), and conflicts were resolved in consultation with the senior author (LT).

**Table 1.** Categorization of the capabilit**y**, opportunit**y**, and motivation (COM-B) components, the theoretical domains framework (TDF) domains with examples of the 9 behavior change wheel (BCW) intervention functions that can be used to influence behavior.

COM-B component	TDF domains	BCW intervention functions
• **Capability** (psychological & physical)	• Knowledge • Skills (cognitive and physical) • Memory, attention, decision processes • Behavioral Regulation	• Education • Training • Enablement • Environmental restructuring • Modeling

• **Opportunity** (physical & social)	• Environmental context & resources • Social influences	• Environmental restructuring • Restriction • Modeling • Enablement

• **Motivation** (reflective & automatic)	• Role & identity • Beliefs (capabilities, consequences) • Optimism • Intentions, goals • Reinforcement • Emotion	• Education • Persuasion • Incentivization • Coercion • Modeling • Enablement

## 3. Results

### 3.1. Study selection

**Figure 1.**
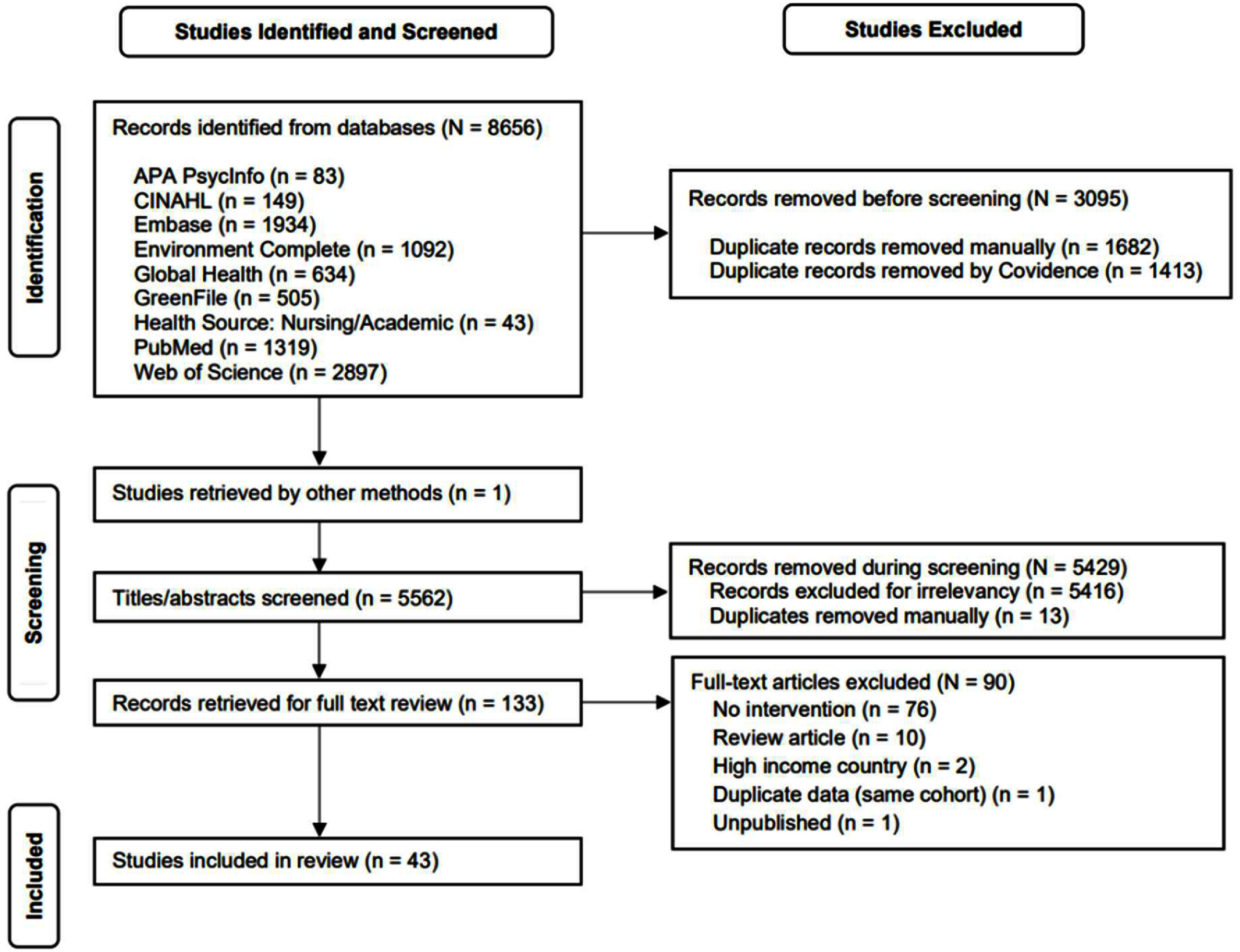
PRISMA flow diagram illustrates identification, screening, eligibility, and inclusion of studies.

A total of 8656 citations from the databases were uploaded to EndNote X20 and 1682 duplicates were manually removed. This left 6974 initially eligible studies that were uploaded to Covidence systematic review software. Covidence identified 1413 additional duplicates, leaving 5561 records. One additional study was from other sources (Buntaine *et al* 2024). Title and abstract screening for eligibility were performed by five independent investigators (HR, KC, AM, VG, LT) according to the inclusion/exclusion criteria. Conflicts between the reviewers were resolved by discussion. Of these records, 5416 were excluded for irrelevancy, and 13 additional duplicates were identified and manually excluded, leaving 133 eligible for full-text review. Of these, 90 were excluded, leaving 43 for data extraction and synthesis. The review and selection processes for the studies are summarized in the PRISMA flow diagram in figure 1.

### 3.2. Study characteristics

A brief inventory of study interventions to address plastic problems is presented in table 2. Supplementary material B provides detailed information extracted from all studies, including study methods and results. Of the 43 included studies, 4 were quantitative randomized controlled trials, 20 were quantitative non-randomized trials, 10 were quantitative descriptive studies, 6 were qualitative, and 3 were identified as mixed methods studies. Two of the quantitative studies had a qualitative component without being mixed-methods studies. More than 50% of the studies (23 out of 43) had no follow-up period and of those that did, the maximum follow-up period was one year (Bharadwaj *et al* 2021).

**Table 2.** Inventory of behavioral interventions used to address plastic waste problems in 43 studies.

Study	Plastic waste problem addressed	Brief description of intervention	Country	Participant age, sample size	Participant gender
Adeboye *et al* (2023)	Lack of awareness about solid waste management	Waste sorting training for students; segregation stands placed in high-traffic areas in schools	Nigeria	8–13 years (*n* = 120)	Men, women

Albach *et al* (2010)	Environmental impacts caused by inadequate disposal of packaging waste	Ecological checkout: bring bag, or sort and leave packaging at supermarkets	Brazil	Not reported (*n* = 350)	Men, women

Ameniabar Cristi *et al* (2020)	Environmental problem posed by single-use plastic bags	Community educational workshop to reduce use of single use plastic bags	Chile	18–76 (*n* = 257)	Men, women

Antinyan and Corazzini (2025)	Excessive use of single use plastic bags given to customers in grocery stores	Environmental nudge, or financial bonus scheme, both with and without a free reusable bag, at chain grocery store	Armenia	Not reported (*n* = 4150)	Not reported

Barros *et al* (2013)	Managing municipal solid waste (MSW) on a university campus	Waste management procedures using onsite composting and waste sorting bins and environmental education	Brazil	Not reported	Men, women

Bharadwaj *et al* (2021)	Increasing plastic waste and environmental pollution caused by single-use plastic bags	Plastic bag ban, including penalties for non-compliance and promotion of reusable bag use. Awareness campaigns, monitoring of retailers, and fines to encourage compliance	Nepal	Not reported (*n* = 108–156)	Not reported

Buntaine *et al* (2024)	Informal waste burning and air pollution in urban areas	After initial education campaign, social competition among villages to stimulate collective action against informal waste burning	Uganda	Not reported	Not reported

Cheng *et al* (2022)	Low recycling participation among youth	Edcraft gamified learning (EGL), an online gamified recycling activity	Malaysia	15–24 years (*n* = 124)	Men, Women

Eduardo *et al* (2023)	Gap in knowledge about environmental impacts of plastic, including production chain, environmental consequences, and waste management	Project-based learning integrating biology and chemistry disciplines to develop students’ environmental awareness about plastic production	Brazil	First year high school students (*n* = 62)	Not reported
Ferronato *et al* (2020)	Insufficient knowledge about recycling processes and benefits of waste valorization; absence of proper waste collection and recycling infrastructure	Selective collection (SC) system for recyclable waste at a university by introducing designated bins for plastics and paper/cardboard and conducting awareness campaigns	Bolivia	University students (*n* = 610)	Men, women

Garcia *et al* (2021)	Marine debris generated by recreational fisheries	Placement of specifically designed collector bins with angler-oriented signage at coastline	Argentina	Not reported (*n* = 22)	Men, women

Hanson (2016)	Increasing amounts of plastic waste, state-sponsored economic development programs target men, excluding women	Formation of women’s recycling group	Mexico	Not reported (*n* = 7)	Women

Huda and Ramadhan (2021)	Lack of awareness about dangers of plastic waste	Educational game to increase environmental awareness	Indonesia	Elementary school children (*n* = 45)	Men, women

Jirapornvaree *et al* (2023)	Plastic waste management and increasing accumulation of plastic waste	Monitored and assessed Thailand roadmap on plastic waste management (2018–30)	Thailand	Not reported (*n* = 2378–survey participants; *n* = 13 interviews)	Not reported

Kittu *et al* (2023)	Hazards of single use plastic and waste management	Single use plastic bag ban	India	Above 18 years (*n* = 450)	Men, Women

Foolmaun *et al* (2021)	Environmental hazards of improper use and disposal of plastic bags	Government ban on import, manufacture, sale, and distribution of plastic bags	Mauritius	Not reported (*n* = 308)	Men, women

Kusnoputranto *et al* (2020)	Enhancing motivation to manage waste sent to landfills	Educational intervention in waste processing for teachers and students	Indonesia	Elementary school students and teachers (*n* = 32)	Men, women

Liu *et al* (2025)	Lack of studies integrating Buddhist spiritual values with practical waste management solutions to manage plastic waste crisis in Thailand	Buddhist temple/community-based plastic waste management system grounded in Buddhist Eco-Sattva principles using 3 R’s (reduce, reuse and recycle)	Thailand	25–60 years (*n* = 15)	Not reported
Manuel *et al* (2015)	Inadequate knowledge regarding hazards of plastic waste and its disposal	Educational interventions to improve awareness and knowledge about dangers posed by plastic waste in rural communities	India	20–45 years (*n* = 100)	Men, women

Mapotse and Mashiloane (2017)	Community and school-based littering	Progressive environmental action research (PEAR) to raise awareness; activities such as celebrating Arbor Day, creating a vegetable garden, and conducting litter pick-up campaigns	South Africa	School youth (*n* = 14)	Not reported

Mathis *et al* (2022)	Ocean plastic pollution	Municipal waste recycling program	Indonesia, Philippines, Sri Lanka, and Vietnam	Not reported but included youth	Men, women

Neef *et al* (2023)	Excessive use of single use plastic bags	Optimistic narrative future vision scenario video of shoppers choosing to use re-usable bags	South Africa	18–29 years (*n* = 342)	Men, women

Oduro-Kwarteng *et al* (2016)	Inadequate waste segregation systems, low recycling rates, and reliance on informal recycling	Educational intervention on separation of waste types, provision of waste bags	Ghana	Not reported (*n* = 60)	Not reported

Olaseha *et al* (2005)	Improper waste management leading to dumped garbage and poor sanitation	Educational program for vegetable market traders	Nigeria	All ages (*n* = 220)	Men, Women

Omondi and Asari (2021)	Environmental and socioeconomic issues posed by single-use plastic bags	Plastic bag ban including penalties for non-compliance and promotion of reusable bag use	Kenya	18 − > 50 years (*n* = 150)	Men, women

Pakasi *et al* (2024)	Inability of government to transport waste has led to accumulating waste in communities	Community-based waste management system in 3 communities, including women-run waste banks to reduce, re-use and recycle	Indonesia	Not reported (*n* = 30 in-depth interviews, 6 focused groups)	Men, women

Qu (2025)	China is among the world’s largest producers of plastic waste	Government ban on plastic straws	China	Not reported (*n* = 204 preliminary questionnaires, *n* = 513 online questionnaires)	Not reported
Raidee *et al* (2024)	Plastic toothbrushes contribute to plastic pollution and cannot be recycled	Bamboo toothbrushes provided, participants asked to use them for 2 weeks; product evaluation	Malaysia	18–40 years (*n* = 10)	Men, women

Raza *et al* (2021)	Plastic waste management and increasing accumulation of plastic waste	Eco-friendly advertising appeals to promote use of bio-nanomaterial plastics among consumers	Pakistan	Above 18 years (*n* = 364)	Men, women

Sandarenu *et al* (2017)	Inefficiencies in current waste management systems	Provision of black polythene bags to households for monthly polythene and plastic waste recycling	Sri Lanka	Not reported (*n* = 100)	Not reported

Sato *et al* (2020)	Ineffective solid waste management and lack of source segregation	Distribution of Household compost bin and awareness of waste separation	Sri Lanka	Not reported (*n* = 317 household surveys; *n* = 117 business surveys; *n* = 7 recycling businesses surveyed)	Not reported

Sedtha *et al* (2023)	Environmental impact and ineffective waste management for single use plastic	Educational campaigns, regulatory measures (bans and fees), and incentives for using alternatives single use plastics	Thailand	Not reported (*n* = 31)	Not reported

Senturk and Dumludag (2021)	Environmental problems caused by disposal of single use plastics	Policy to impose plastic bag fee	Turkey	>18 years (*n* = 789)	Men, Women

Severin *et al* (2023)	Drastic increase in production and release of municipal solid waste on the coasts	Trained students from secondary schools in sampling and analyzing macro-, meso- and microplastics found on sandy beaches	Benin, Cabo Verde, Côte d’Ivoire, Ghana, Morocco, Nigeria and Malaysia	11–22 years (*n* = 410)	Not reported

Simmons and Sanders (2022)	Plastic waste pollution and threat to marine life due to fishing practices	Educational meetings, face-to-face	Indonesia and Philippines	Not reported	Men, women

Sofia *et al* (2021)	Inadequate waste management at household level	Provision of plastic bags, education and coaching for waste separation	Indonesia	Housewives (not reported) (n = 100)	Females
Spranz *et al* (2018)	Increased plastic pollution	Use of social norms, indirect monetary incentives or authority endorsement to reduce use of plastic bags	Indonesia	Not reported (*n* = 74 survey respondents)	Men, women

Tran *et al* (2020)	Poor management of increasing amounts of solid waste, including plastic	Waste separation at source program	Vietnam	Not reported (*n* = 402)	Not reported

Uneputty *et al* (1998)	Litter pollution on shores	Shore cleanup	Indonesia	Not reported (*n* = 115)	Men, women

YalvaÇ *et al* (2023)	Increased demand for single use plastic bags has an environmental impact due to lack of recycling	“Plastic bags law” prohibits free provision of plastic bags >15 *μ*ms to customers	Turkey	18 − > 50 years (*n* = 1537)	Men, women

Yelvattimath *et al* (2014)	Low knowledge levels among rural women regarding health and environmental issues	Community radio educational programs on environmental health topics, including 3 Rs	India	Not reported (*n* = 120)	Women

Zhao *et al* (2023)	Extensive use of plastic film mulching which does not degrade in soil poses a risk for environmental pollution	Mandatory replacement of plastic film mulch recycling policy—farmers replace old waste films for new film	China	30–69 years (*n* = 200)	Men, women

Zhou *et al* (2021)	Inefficient resource allocation in current recycling systems; unsophisticated recycling information platforms	Incentive-based waste recycling system using Internet of Things (IoTs) and data analytic technologies to provide price adjustments, forecast waste collection amounts, and facilitate information sharing among stakeholders	China	Not reported	Not reported

### 3.3. Risk of bias in studies

**Figure 2.**
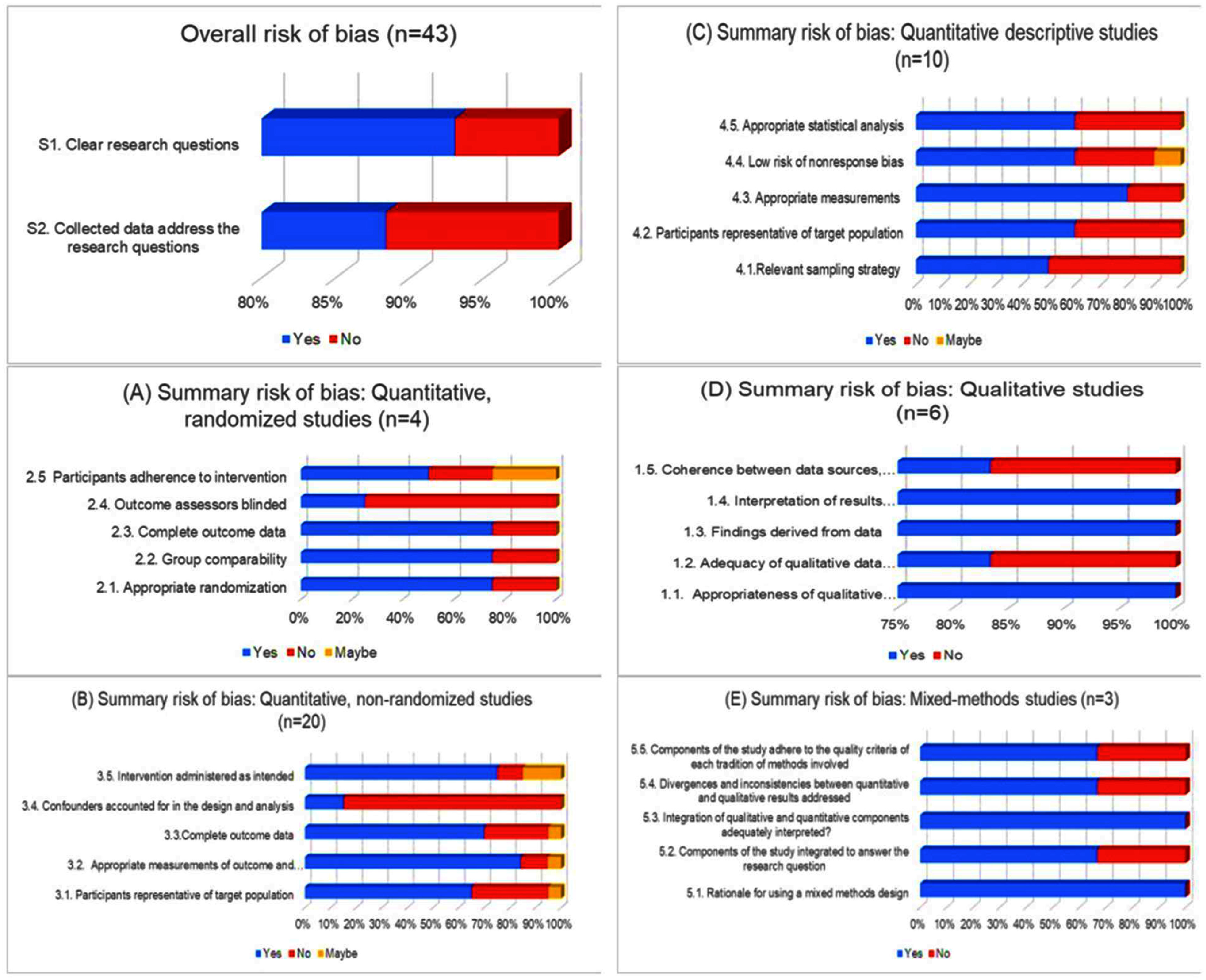
Summary of risk of bias using the mixed methods appraisal tool (MMAT).

Using the MMAT, studies were assessed for potential bias and methodological quality. Among the 43 studies screened, 38 (90.5%) passed both initial MMAT screening questions, indicating they had clearly stated research questions and collected data that aligned with those objectives. The remaining four studies lacked either clearly defined questions or sufficient data to answer them. Many quantitative descriptive studies did not use appropriate statistical methods in the analysis (40%) and most non-randomized studies did not include confounders in the analyzes (85%). Figure 2 provides a detailed visual illustration of the MMAT criteria across different study designs. The MMAT discourages the exclusion of studies with low methodological quality; therefore, we included the full range of studies for analysis, described in supplementary material C.

### 3.4. Population characteristics

The 43 included studies represented interventions from 27 unique countries across Asia, Africa, and Latin America, highlighting both geographic diversity and relevance to LIC, LMIC, and UMIC contexts. Youth were the focus in several school-based interventions (Huda and Ramadhan 2021, Adeboye *et al* 2023, Severin *et al* 2023), while women were specifically targeted in community-led waste initiatives (Hanson 2016, Sofia *et al* 2021, Pakasi *et al* 2024). Other studies addressed broader community engagement involving households (Sandarenu *et al* 2017, Sato *et al* 2020), market vendors (Olaseha *et al* 2005), both educators and students (Kusnoputranto *et al* 2020, Eduardo *et al* 2023), and specific occupations like fishermen (García *et al* 2021, Simmons and Sanders 2022).

### 3.5. Categorization of interventions

The range of strategies and interventions targeted at changing behaviors related to plastic waste management employed across the 43 studies can be broadly categorized and synthesized as follows:

#### 3.5.1. Educational and awareness campaigns

Most interventions relied on education as a foundational component, delivered through schools, community meetings, media, and posters (Olaseha *et al* 2005, Yelvattimath *et al* 2014, Manuel *et al* 2015, Oduro-Kwarteng *et al* 2016, Amenábar Cristi *et al* 2020, Kusnoputranto *et al* 2020, Simmons and Sanders 2022, Adeboye *et al* 2023, Eduardo *et al* 2023, Sedtha *et al* 2023, Severin *et al* 2023).

#### 3.5.2. Community-based and participatory programs

Some studies adopted grassroots or participatory models, including environmental action groups, waste banks (community-based organizations where residents, often women, sort, clean, and deposit various types of waste, especially plastics, to be sold for recycling, promoting waste reduction, reuse, and recycling while generating small incomes), and peer-led community cleanups or healthy social competitions to improve eco-friendly practices (Uneputty *et al* 1998, Buntaine *et al* 2024, Pakasi *et al* 2024). One study used a shore cleanup activity as a means to reduce plastic waste in communities (Uneputty *et al* 1998).

#### 3.5.3. Infrastructure and environmental restructuring

Several studies modified the environment through the provision of collection bins, composting facilities, or organized waste segregation systems (Barros *et al* 2013, Sandarenu *et al* 2017, Ferronato *et al* 2020, Sato *et al* 2020, Tran *et al* 2020, García *et al* 2021, Sofia *et al* 2021). One study provided bamboo toothbrushes as a means to reduce plastic toothbrush waste (Raidee *et al* 2024). One study examined the impact and user experiences of national plastic straw ban (Qu 2025).

#### 3.5.4. Policy and regulatory measures

Several studies examined the effect of plastic bag bans, mandatory waste segregation policies, municipal recycling programs, and policy enforcement (Bharadwaj *et al* 2021, Foolmaun *et al* 2021, Omondi and Asari 2021, Senturk and Dumludag 2021, Mathis *et al* 2022, Jirapornvaree *et al* 2023, Kittu *et al* 2023, YalvaÇ *et al* 2023, Qu 2025).

#### 3.5.5. Economic incentives and disincentives

Four studies examined financial rewards, the provision of free shopping bags, job creation, discounts, or penalties for non-compliance (Foolmaun *et al* 2021, Zhou *et al* 2021, Zhao *et al* 2023, Antinyan and Corazzini 2025).

#### 3.5.6. Gamification and digital tools

Four studies used mobile phone applications or interactive game activities to foster behavior change (Huda and Ramadhan 2021, Raza *et al* 2021, Cheng *et al* 2022, Neef *et al* 2023).

#### 3.5.7. Social norms and behavioral nudges

Some programs leveraged role models, religious frameworks, or collective identity to promote eco-friendly practices (Albach *et al* 2010, Spranz *et al* 2018, Antinyan and Corazzini 2025, Liu *et al* 2025).

#### 3.5.8. Community-led, gender-specific interventions

Two studies leveraged women as agents of change to drive local solutions to environmental problems (Hanson 2016, Pakasi *et al* 2024).

### 3.6. BCW intervention functions

Among the BCW intervention functions used to address plastic waste, *education* was the most frequently employed and was used in approximately 76.7% of the studies (*k* = 33). Education primarily targeted increased awareness regarding the harms of plastic waste and targeted knowledge change about available alternatives. Education was delivered through school curricula (Yelvattimath *et al* 2014, Huda and Ramadhan 2021), community workshops (Manuel *et al* 2015, Raza *et al* 2021), and digital platforms (Cheng *et al* 2022).

*Environmental restructuring*, used in 30 out of 43 studies (69.8%), focuses on modifying the physical environment to enable proper plastic disposal or substitution. Examples include the introduction of public waste sorting bins (Ferronato *et al* 2020), waste bank systems (Pakasi *et al* 2024), and redesigned market infrastructure (Barros *et al* 2013). *Incentivization* was another frequently used intervention function (*k*= 21), entailing direct and indirect financial incentives such as job creation, low-cost bulk fertilizers, subsidies, and direct financial bonuses (Olaseha *et al* 2005, Sato *et al* 2020, Sedtha *et al* 2023), as well as non-financial incentives such as certificates of appreciation or grading from teachers (Ferronato *et al* 2020, Eduardo *et al* 2023, Buntaine *et al* 2024). *Persuasion* was used in 46.5% of the studies (*k* = 20), often involving emotional appeals about the harmful consequences of plastic pollution (Neef *et al* 2023, Liu *et al* 2025). *Training and enablement* were present in 15 and 9 studies, respectively. *Training* targeted skills development such as sorting waste or using eco-alternatives to dispose of waste (Kusnoputranto *et al* 2020, Adeboye *et al* 2023). *Enablement* interventions included logistical support and community mobilization (García *et al* 2021, Sofia *et al* 2021) (see table 3). *Modeling* (*k*= 9) was used in interventions that leveraged influential individuals (e.g. community leaders, teachers, monks) to demonstrate desired behaviors (Mapotse and Mashiloane 2017, Liu *et al* 2025). *Coercion* (*k*= 5) was less commonly used as an intervention strategy and was generally applied through enforcement of bans or fines (Bharadwaj *et al* 2021, Foolmaun *et al* 2021, Omondi and Asari 2021, Sedtha *et al* 2023, Qu 2025). *Restriction* mainly appeared as banning certain products like plastic bags or straws (Kusnoputranto *et al* 2020, García *et al* 2021, Sofia *et al* 2021, Adeboye *et al* 2023, YalvaÇ *et al* 2023, Qu 2025).

**Table 3.** Components of behavioral interventions mapped to behavior change wheel (BCW) intervention functions.

Study	Education	Persuasion	Incentivization	Coercion	Training	Restriction	Environmental restructuring	Modeling	Enablement
Adeboye *et al* (2023)	X				X		X		
Albach *et al* (2010)	X				X		X		
Ameniabar Cristi *et al* (2020)	X	X	X				X		
Antinyan and Corazzini (2025)	X	X	X				X		X
Barros *et al* (2013)	X	X			X		X		
Bharadwaj *et al* (2021)	X	X		X		X			
Buntaine *et al* (2024)	X		X				X		X
Cheng *et al* (2022)	X		X						
Eduardo *et al* (2023)	X		X		X				
Ferronato *et al* (2020)	X	X	X				X	X	
García *et al* (2021)	X	X					X		
Hanson (2016)	X	X					X	X	X
Huda and Ramadhan (2021)	X	X	X		X				
Jirapornvaree *et al* (2023)	X					X	X		X
Kittu *et al* (2023)						X	X		
Foolmaun *et al* (2021)		X		X		X			
Kusnoputranto *et al* (2020)	X		X		X		X	X	
Liu *et al* (2025)	X	X	X		X		X	X	
Manuel *et al* (2015)	X								
Mapotse and Mashiloane (2017)	X	X	X		X		X		X
Mathis *et al* (2022)	X	X	X		X		X		
Neef *et al* (2023)		X						X	
Oduro-Kwarteng *et al* (2016)	X						X		
Olaseha *et al* (2005)	X	X	X				X	X	
Omondi and Asari (2021)				X		X			
Pakasi *et al* (2024)	X		X		X		X		X
Qu (2025)				X		X	X		
Raidee *et al* (2024)							X		
Raza *et al* (2021)	X								
Sandarenu *et al* (2017)							X		
Sato *et al* (2020)	X		X		X		X		
Sedtha *et al* (2023)	X	X	X	X		X	X	X	
Senturk and Dumludag (2021)		X	X			X			
Severin *et al* (2023)	X				X				X
Simmons and Sanders (2022)	X		X		X		X	X	
Sofia *et al* (2021)	X						X		
Spranz *et al* (2018)	X	X	X				X		X
Tran *et al* (2020)	X	X	X				X		
Uneputty *et al* (1998)	X	X	X				X	X	
YalvaÇ *et al* (2023)						X			
Yelvattimath *et al* (2014)	X	X			X				
Zhao *et al* (2023)	X		X				X		
Zhou *et al* (2021)			X		X		X		X

Legend: X = intervention function addressed in intervention; blank = not addressed.

### 3.7. COM-B model and TDF domains

The choice of intervention functions is dependent upon the behaviors that are targeted for change. The COM-B model and the TDF domains were used to categorize the sources of these behaviors in the reviewed studies. Overall, most interventions addressed *physical and psychological capability* along with *physical opportunity. psychological capability* was frequently targeted through the TDF domains of *knowledge* (*k*= 36) and *skills* (*k* = 20) with many interventions aiming to increase awareness about plastic waste, proper disposal, and recycling techniques, but *memory, attention, and decision processes* (*k* = 3) *and behavioral regulation* (*k* = 3) were rarely used. *Physical opportunity* includes *environmental context and resources,* one of the most targeted TDF domains. It was addressed in 37 studies, largely through the provision of physical tools and infrastructure. *Social opportunity* was addressed through the TDF domain of *Social Influences* (*k* = 24) often by providing peer support, community leader involvement, and collective clean-up events. There was less emphasis on *reflective and automatic motivation. reflective motivation* was targeted primarily through *beliefs about capabilities* (*k*= 19), *intentions* (*k*= 16), *social/professional role and identity* (*k* = 13), *goals* (*k*= 11), *belief about consequences* (*k* = 7) and *optimism* (*k* = 4).TDF domains related to *automatic motivation* including *emotion* (*k* = 11), and *reinforcement* (*k* = 15) were less commonly used. (See table 4).

**Table 4.** Components of behavioral interventions mapped to the theoretical domains framework and the COM-B framework.

Study	CAPABILITY				OPPORTUNITY		MOTIVATION							
	Physical	Psychological			Physical	Social	Reflective						Automatic	
	Skills	*K*	Mem-AttDec	BehReg	EnvRes	SoInf	SoPro	BelCap	Opt	BelCon	Intent	Goals	Reinf	Emo
Adeboye *et al* (2023)		X	X		X			X						
Albach *et al* (2010)	X	X			X			X			X			
Amenábar Cristi *et al* (2020)		X			X	X						X		
Antinyan and Corazzini (2025)		X			X							X	X	
Barros *et al* (2013)	X	X		X	X		X					X	X	
Bharadwaj *et al* (2021)		X			X					X			X	
Buntaine *et al* (2024)		X				X	X	X		X		X		X
Cheng *et al* (2022)	X	X				X					X	X		X
Eduardo *et al* (2023)		X	X			X		X				X		
Ferronato *et al* (2020)	X	X			X	X	X	X						
García *et al* (2021)		X			X		X			X				X
Hanson (2016)	X	X			X	X	X	X			X	X	X	X
Huda and Ramadhan (2021)	X	X				X								X
Jirapornvaree *et al* (2023)		X			X	X					X			
Kittu *et al* (2023)					X									
Foolmaun *et al* (2021)		X			X								X	
Kusnoputranto *et al* (2020)	X	X			X	X	X	X						
Liu *et al* (2025)	X	X			X	X	X	X		X	X	X		
Manuel *et al* (2015)		X				X								
Mapotse and Mashiloane (2017)	X	X			X	X	X	X	X		X	X	X	X
Mathis *et al* (2022)	X	X			X	X		X						
Neef *et al* (2023)				X				X	X		X			X
Oduro-Kwarteng *et al* (2016)	X	X			X								X	
Olaseha *et al* (2005)	X	X			X	X	X	X		X			X	X
Omondi and Asari (2021)					X								X	
Pakasi *et al* (2024)	X	X			X	X	X	X	X	X	X	X		
Qu (2025)		X			X								X	
Raidee *et al* (2024)					X	X					X			
Raza *et al* (2021)		X			X			X			X		X	
Sandarenu *et al* (2017)					X									
Sato *et al* (2020)	X	X			X									
Sedtha *et al* (2023)		X			X	X		X			X		X	X
Senturk and Dumludag (2021)					X		X							
Severin *et al* (2023)	X	X			X			X		X	X			X
Simmons and Sanders (2022)	X	X	X		X	X		X	X			X		X
Sofia *et al* (2021)	X	X			X									
Spranz *et al* (2018)		X			X	X	X	X						
Tran *et al* (2020)		X			X	X	X	X			X		X	
Uneputty *et al* (1998)	X	X			X	X					X			
Yalvac *et al* (2023)					X									
Yelvattimath *et al* (2014)	X	X			X	X								
Zhao *et al* (2023)		X		X	X	X					X		X	
Zhou *et al* (2021)		X			X	X					X		X	

Legend: X = domain addressed in intervention; blank = not addressed; K = knowledge; MemAttenDec = memory, attention, and decision processes; BehReg = behavioral regulation; EnvRes = environmental context and resources; SoInf = social influences; SoPro = social/professional role and edentity; BelCap = beliefs about capabilities; Opt = optimism; BelCon = beliefs about consequences; Intent = intentions; Goals = goals; Reinf = reinforcement; Emo = emotion.

## 4. Discussion

This review identified 43 studies related to interventions that targeted reducing plastic waste in low-resource countries. These interventions were systematically mapped to the BCW intervention functions, the COM-B components and the TDF domains. The 43 studies represented interventions from 27 unique countries across Asia, Africa, and Latin America, highlighting both geographic diversity and relevance to low-resource contexts. Study populations were diverse and included a range of demographic groups and stakeholders. Across all of the studies, the most common interventions can be broadly synthesized into educational campaigns; community-based programs; environmental modification; policy changes, including incentives; behavioral nudges; and gender-specific interventions.

Intervention approaches varied widely, and included school-based campaigns, policy-led plastic bans, digital gamification tools, waste bank systems, and community clean-up programs. Many interventions were implemented at the municipal or grassroots level, while others were linked to national policies or multi-stakeholder collaborations. Most interventions focused on single use plastic, particularly plastic bags and packaging. A few, however, expanded to broader categories of plastic pollution, including plastic toothbrushes (Raidee *et al* 2024), plastic mulch films in agriculture (Zhao *et al* 2023), and marine plastic debris (García *et al* 2021). A small number leveraged community leadership (Spranz *et al* 2018, Buntaine *et al* 2024) and religious principles (Liu *et al* 2025) to spread messaging related to the reduction in plastic waste. These findings underscore the wide range of both the behavioral targets and intervention mechanisms employed. A point to be noted is that while many community groups were engaged, few interventions actually engaged municipal or other community leaders, instead relying on other groups such as teachers, farmers, women and other grass roots organizations.

### 4.1. Dominance of education as an entry point

Educational approaches were overwhelmingly favored across all geographies and demographics. While this suggests a global consensus on the importance of knowledge and raising awareness, the reliance on education alone may not be enough to motivate behavior change (Ferris *et al* 2001). For instance, short-term interventions using only radio broadcasts or school lessons, without accompanying structural or motivational strategies, may not lead to long-term change. It is well documented that reliance on only education for any behavior change, such as medication adherence (Hennessey and Heryer 2011), or handwashing (Zohura *et al* 2020) is not sufficient. While educational approaches appear to have been employed most frequently, likely due to their relative ease of implementation, the long-term outcome of these interventions, particularly when not combined with other intervention functions, remains uncertain.

### 4.2. Frequent use of multi-modal interventions

Several programs combined multiple BCW intervention functions, particularly education, environmental restructuring, and enablement. For example, initiatives in Indonesia and Ghana included waste separation education alongside the provision of sorting bins and visible role models (Oduro-Kwarteng *et al* 2016, Kusnoputranto *et al* 2020). These multi-component strategies demonstrated knowledge gain, increased capability, and created opportunities for behavior change. Similarly, Antinyan and Corazzini incorporated incentivization through financial bonuses alongside education and environmental restructuring by providing tote bags (2025). Overall, interventions employing multiple functions appeared to be more frequently employed than those relying solely on education (Gordon *et al* 2001). This could be because employing multimodal strategies has been shown to be more effective than education only. A recent systematic review and meta-analysis of 24 studies evaluating waste management education among healthcare workers found that educational interventions, particularly multicomponent approaches combining didactic instruction, hands on training, system changes, and reminders, consistently improved both knowledge and waste handling practices (Gordon *et al* 2001). Notably, Gordon’s review reported that multicomponent interventions that included education produced substantially larger gains than single component educational approaches. Although educational interventions are often the first strategy employed to improve practices, education alone may be insufficient to produce meaningful or sustained behavior change. This underscores the importance of systematically mapping intervention functions to frameworks such as COM-B and the BCW, to identify the combinations that can maximize impact and sustainability in low-resource settings.

### 4.3. Incentives and coercion as emerging levers

One of the commonly implemented approaches used incentives (e.g. financial bonuses, job creation, competitions) and coercive elements (e.g. bans, fines). The latter was most often applied in policy-led initiatives. For example, national-level plastic bag ban in Nepal, when combined with public awareness campaigns, monitoring, and penalties, led to reduction in single-use plastic usage (Bharadwaj *et al* 2021). While this example offers a useful lesson, its applicability is not universal; the success of interventions depends on tailoring interventions to local social, cultural, and infrastructural contexts. Indeed, many studies noted that stand-alone policy interventions were insufficient: Kittu *et al* found that more than half of the participants viewed the plastic bag ban unfavorably (2023); and in Yalvac *et al*, since the law only prohibited the free distribution of plastic bags >15 *μ*ms, there was an increase in the use of thinner bags (⩽15 *μ*ms) that remained free (2023). In the past, incentives and coercion have been used to exert policy change, as well as in public health campaigns. Evidence from tobacco control demonstrates that incentives and coercive policy measures, such as financial rewards (Volpp *et al* 2009), taxation (Chaloupka *et al* 2011), and regulatory restrictions (Lupton and Townsend 2015), have been central to achieving sustained behavior change. These approaches operate by reshaping motivation, opportunity, and social norms, suggesting that similar strategies can be useful for plastic waste management, especially in low-resource settings.

### 4.4. Underutilization of emotion and identity-based strategies

Few interventions engaged emotional responses or leveraged individual or group identity. Where used, such as in South Africa’s optimistic narrative video (Neef *et al* 2023) or Thailand’s integration of Buddhist teachings (Liu *et al* 2025), these approaches showed potential in shifting deeper motivational states. Similarly, important TDF domains such as beliefs about consequences, goals, and intentions were infrequently targeted, despite their potential to strengthen internal motivation and align behaviors with long-term values. Addressing these domains is critical, as they are more likely to foster sustainable changes in behavior compared to interventions that focus only on knowledge provision. Past examples of interventions that resonate with identity (Oyserman *et al* 2014, Steffens *et al* 2021) or cultural contextuality (Herman *et al* 2025) have yielded noticeable results. Emotion is a fundamental human factor that shapes risk perception, motivation, and decision-making. It can be a powerful, yet often underutilized, leverage point in public health. Studies show that emotionally salient messaging emphasizing harm to children, as well as identity-based appeals tied to being a responsible parent, are stronger predictors of correct safety practices than awareness of guidelines alone (Morrongiello *et al* 2014, Will *et al* 2015). Future programs could benefit from embedding emotional resonance and cultural relevance into intervention design and delivery.

### 4.5. Gender and social equity gaps

Despite the significant role women play in household waste management, few interventions explicitly targeted or sought to empower them. Two specific studies aimed to empower women through the use of women-led waste banks in Indonesia and recycling groups in Mexico (Hanson 2016, Pakasi *et al* 2024). However, it is important to recognize that in some contexts, women may experience negative impacts from the uneven burden of waste sorting, management, and recycling responsibilities, rather than feeling empowered. Integrating a gender lens and promoting equitable participation could enhance both inclusiveness and impact. Given that women play a major role in sustaining daily household activities, targeting women at the grassroots level by educating and incentivizing them to adopt the principles of reduce, reuse, and recycle could serve as a practical and impactful entry point for improving waste management practices (Moeini *et al* 2024).

### 4.6. Sustainability and long-term impact

Many studies used short-term strategies as interventions such as beach clean-ups, social competitions, bamboo toothbrushes, and educational programs. A major limitation across studies was the lack of longitudinal follow-up. The sustainability of such interventions is thus questionable. Bharadwaj *et al* was the only study in our review with a follow-up period of one year (Bharadwaj *et al* 2021). Most studies were cross-sectional in nature and did not conduct follow-up after the intervention. Without reinforcement mechanisms (e.g. ongoing support, community recognition), initial gains may not translate into long-term behavioral shifts.

### 4.7. Dearth of literature on behaviors to reduce plastic waste burning

Open burning of plastic waste is a significant global issue, particularly in low-resource countries, where inadequate waste management infrastructure leads to widespread informal disposal practices. Approximately 40%–65% of municipal solid waste in low-resource countries is disposed of through open burning, posing serious risks to public health and the environment (Velis and Cook 2021). Only one intervention study in this review focused on waste burning, using a social competition to reduce informal waste burning in Uganda (Buntaine *et al* 2024). The intervention led to a 24% reduction in waste burning in treated neighborhoods compared to controls, with sustained effects observed several months after the competition. It is essential to understand the importance of context-specific, multi-faceted interventions in addressing the complex issue of plastic waste burning. While strategies such as social competition and community-based education have shown promise, their effectiveness can be enhanced when tailored to local cultural and infrastructural contexts.

### 4.8. Shift in literature over time

While undertaking this review, we came across many recent studies that utilized statistical modeling (Edoria *et al* 2023, Zahid *et al* 2025) and evaluated willingness to pay for plastic waste management (Chatterjee and Barbhuiya 2021, Tyllianakis and Ferrini 2021). These were based on hypothetical scenarios used to predict future behavior change related to plastic waste management. Because these studies were hypothetical, and not implemented, they were excluded from our review. Studies focused on the potential for replacing plastic with eco-friendly alternatives like the use of biofilms or biodegradable cutlery as a means to reduce plastic waste, but did not implement an intervention (Battulga *et al* 2024, Tudu and Mishra 2025). Recent studies focused on citizen science, where participants engaged in data collection to enumerate plastic waste, without examining interventions to reduce plastic waste (Ribeiro *et al* 2021, Nguyen *et al* 2022). We also observed a trending shift towards a focus on measuring knowledge about microplastics in the environment (Maharjan 2024, Pratiwi *et al* 2024). While these strategies are effective for raising awareness, their impact on actual behavior change remains limited, highlighting the need for interventions that go beyond knowledge acquisition.

### 4.9. Future directions

Many of the reviewed studies used education as a mechanism for behavior change but did not conduct long-term follow-up. Education is a fundamental component for behavior change, but there is a consensus in scientific literature that it is insufficient (Arlinghaus and Johnston 2017, Social and behavior change | UNICEF n.d.). Future studies should consider coupling education with other intervention functions that address opportunities and motivations, important components of sustained behavior change.

Low-resource countries struggle with plastic trash piling up on roadsides and beaches, or the burning of trash in open fires. As a method to address this situation, several studies provided bins or bags for trash disposal. While these approaches may facilitate proper disposal and sorting of plastic waste, in the long term they do not reduce the amount of waste that is generated (Trushna *et al* 2024). We recommend coupling waste collection and sorting activities with education targeted at reducing the use of, or the refusal to use, plastic.

Few studies used creative strategies like healthy competitions among neighborhoods or leveraging religious principles to instill behavior change related to plastic waste. Such strategies targeted intrinsic motivation among participants, which may in turn help sustain the achieved change. Our study group has undertaken the Ecolectivos intervention trial in rural Guatemala to address plastic waste burning. This community-based trial employed a combination of education, environmental restructuring, and incentivization to reduce household plastic waste burning (Thompson *et al* 2025).

We found that policy interventions such as bans on single-use plastics were among the most commonly implemented strategies to reduce plastic waste. However, the extent to which such policies lead to sustained behavior change remains uncertain. Humans are creatures of convenience, often gravitating toward readily available options, such as single use plastics, that simplify daily life. For these bans to be effective, governments must ensure that practical and acceptable alternatives are available prior to enforcement. For instance, when plastic straws are prohibited, many individuals resist switching to paper straws due to their fragility and impact on taste (Qu 2025). The provisions of more acceptable substitutes, such as bamboo or metal straws, can improve compliance and public acceptance. The same principle applies to plastic bags: providing free, durable, and reusable bags as an alternative may increase the likelihood of policy success (Antinyan and Corazzini 2025). Ultimately, aligning policy enforcement with accessible alternatives can facilitate a smoother transition and enhance the long-term impact of single use plastic reduction efforts.

Although extensive system and policy changes are necessary for reducing the burden of plastic waste accumulation in the environment, individual and community-based behavioral interventions play a crucial role in addressing sustainable waste management. An important step in reducing plastic waste is the move towards a circular plastics economy where plastic materials can be reused, repurposed, and recycled rather than discarded. This move requires more than systemic changes, it requires a fundamental shift in the individual and community’s behaviors towards plastic waste (Allison *et al* 2022, Pottinger *et al* 2024). Although technological and infrastructural improvements are necessary, the lack of evidence-based behavioral frameworks in many strategies may limit their impact. This highlights the need to integrate behavior-focused approaches alongside systemic solutions to achieve meaningful, long-term reductions in plastic waste.

### 4.10. Limitations and strengths

To the best of our knowledge, the present review is the first to systematically summarize behavioral change interventions to reduce plastic waste in low-resource settings. This review is also the first to apply the BCW, COM-B and the TDF to map interventions targeting plastic waste management behaviors. This enhanced behavioral framework allows for structured, theory-informed insights into behavior change mechanisms. The review synthesizes qualitative, quantitative, and mixed-methods studies, drawing from a wide range of target populations—from women-led community groups to schoolchildren, market vendors, and fishermen. Finally, we offer future directions to support the acceptability and sustainability of those interventions.

While our review has provided important insights into the topic, there are a few limitations. Only peer-reviewed articles in the English language were included. Gray literature was not included. Some of the early grassroots interventions reviewed here were not included in peer-reviewed journals and used simple descriptive statistics to report findings. Many studies did not explicitly report participant demographics such as age and gender. The identification of BCW intervention functions and TDF domains involved an element of subjectivity. Although at least two reviewers met to reach consensus, the process inherently relied on interpretation and may be influenced by our biases. A gray literature review focusing on voluntary single use plastic reduction interventions is recommended to extend our understanding of behavioral interventions. Lastly, this review did not include a meta-analysis or any standardized methods to assess outcomes. Instead, behavior change intervention strategies are summarized narratively based on author reports.

## 5. Conclusions

This systematic review provides a comprehensive, theory-driven synthesis of behavioral interventions aimed at reducing plastic waste in low-resource countries. By applying the BCW, COM-B model and the TDF domains, the review characterizes how interventions across different settings are structured to target behavioral determinants of plastic waste.

Education emerged as the most frequently used intervention function, often forming the foundation for awareness-raising and knowledge-building. However, the findings suggest that pairing education with multiple behavior change functions, such as environmental restructuring, enablement, and incentivization, reflect a more comprehensive approach to addressing the complex behavioral determinants of plastic waste management. Notably, gender-responsive, community-led interventions such as women-led collective organizing efforts may demonstrate promising pathways for embedding sustainable practices at the grassroots level. The review also highlights a lack of long-term follow-up data and limited integration of gender, equity, and sociocultural factors in intervention design and evaluation. To accelerate progress on plastic waste reduction, future interventions should adopt a more holistic and context-sensitive approach with special emphasis on the sustainability of those interventions.

## Data Availability

All data that support the findings of this study are included within the article (and any supplementary files). Supplement A available at https://doi.org/10.1088/1748-9326/ae49a3/data1. Supplement B available at https://doi.org/10.1088/1748-9326/ae49a3/data2. Supplement C available at https://doi.org/10.1088/1748-9326/ae49a3/data3.
